# Mediating Effect of Turnover Intention on the Relationship Between Job Burnout and Quiet Quitting in Nurses

**DOI:** 10.1111/jan.16807

**Published:** 2025-02-10

**Authors:** İbrahim Gün, Feyza Çetinkaya Kutun, Selma Söyük

**Affiliations:** ^1^ Department of Health Management, Faculty of Health Sciences Batman University Batman Turkey; ^2^ Ministry of Health Sultan 2. Abdulhamid Han Training and Research Hospital Istanbul Turkey; ^3^ Department of Health Management, Faculty of Health Sciences İstanbul University‐Cerrahpasa Istanbul Turkey

**Keywords:** job burnout, nurse, nursing, quiet quitting, turnover intention

## Abstract

**Aim:**

This study aimed to investigate the potential mediating role of turnover intention in the relationship between job burnout and quiet quitting among nurses and shed light on the associations between job burnout, turnover intention and quiet quitting intention.

**Design:**

This study was designed as a descriptive, cross‐sectional study.

**Methods:**

A total of 317 nurses were selected using convenience sampling approach from a training and research hospital in Turkey. Quiet quitting, job burnout and turnover intention data were collected using the self‐reported questionnaires using paper‐and‐pencil versions. Pearson correlation analysis, independent sample *t*‐test and mediation analysis was conducted with Process v4.3.

**Results:**

Statistically significant associations among job burnout, turnover intention and quiet quitting were found (*p* < 0.05). Job burnout had a positive effect on turnover intention (*β* = 0.339, *p* < 0.001) and quiet quitting (*β* = 0.245, *p* < 0.001). Additionally, turnover intention had a positive and significant effect on quiet quitting intention of nurses (*β* = 0.336, *p* < 0.001). Moreover, mediation analysis revealed that the association of job burnout with quiet quitting was partially mediated by turnover intention (*β* = 0.034, 95% CI [0.019, 0.054]).

**Conclusion:**

This study enrich our understanding of the associations among study variables and suggest that focusing solely on job burnout without considering the mediating effects of turnover intention might not be adequate for reducing the quiet quitting intention among nurses.

**Impact:**

This study shed light on how job burnout and turnover intention of nurses affect their quiet quitting intention. It has been proven that turnover intention is a significant factor in the relationship between job burnout and quiet quitting. These findings could provide guidance for managers in the administration of nurses.

**Patient or Public Contribution:**

No patient or public contribution.

## 1 Introduction

COVID‐19 has caused significant changes in business life. After the devastating effect of the pandemic was left behind, the concepts of the great resignation and quiet quitting emerged for the first time (Anand et al. 2023). The spread of remote working culture during the pandemic has caused problems such as the blurring of the boundaries between work and family spheres. Then, when demands to return to work began, disappointment and disengagement began on the part of the employees (Aydin and Azizoğlu 2022; Scheyett 2023). Since March 2021, with the Great Resignation, workers in many sectors have started voluntarily quitting their jobs (Formica and Sfodera 2022). Quitting job was also triggered unexpected effects for individuals, organisations, and entire nations (Serenko 2023). Among these unexpected outcomes, perhaps the most striking was the strong resonance of quiet quitting behaviour among employees.

Quiet quitting could be described as ‘one does not literally quit one's job, but rather simply does the work that is expected of the position, without going above and beyond what is expected’ (Scheyett 2023). In other words, while the individual does not leave their job, they put forth only minimal effort, refraining from additional responsibilities or extra initiative; they fulfil solely the duties required by their job description. Formica and Sfodera (2022) defined the term quiet quitting refers to ‘the limited commitment of employees to carry out the assigned duties and to relinquish from any other task not specified in their job description’. Definitions of the concept of quiet quitting generally converge on the idea that employees, rather than leaving their jobs, continue working with minimal effort, doing just enough to avoid termination while refraining from putting in any additional effort. Quiet quitting is such a prevalent behaviour that a recent Gallup study of 15,000 workers aged 18 and older revealed that half of the US workforce consists of quiet quitters (Zuzelo 2023).

COVID‐19 has brought many challenges to working life and especially healthcare workers faced difficulties in work life. Following the COVID‐19 pandemic, remote work gained global popularity. As pandemic effects waned, many US employees resisted returning to workplaces, initiating the ‘Great Resignation’ (Formica and Sfodera 2022). Those unable to leave due to low retirement benefits, job insecurity and challenging work conditions have instead adopted quiet quitting (Formica and Sfodera 2022; Zuzelo 2023). Thus, quiet quitting emerged as an alternative for individuals intending to leave their jobs but unable to do so (Anand et al. 2023; Scheyett 2023). Therefore, understanding the connections between job burnout, turnover intention, and quiet quitting will be a critical step in determining intervention strategies aimed at helping them to cope with work life challenges in nurses. Moreover, measuring quiet quitting is very useful in reducing the lack of commitment to work, protecting and developing the healthcare workforce, facilitating the work of human resources managers, improving the quality of the service provided by nurses and gaining competitive advantage.

The impact of quiet quitting on the organisation is also related to patient care, as a decline in nurses' performance can result in failing to meet patients' needs and inadequate care (Galanis, Moisoglou, et al. 2024). Some studies have already shown that nurses do not have enough time to meet patients' needs during their shifts (Cho et al. 2020) and this situation may be further exacerbated by quiet quitting. Additionally, the widespread occurrence of quiet quitting among nurses presents a significant threat to the healthcare system, given their essential role in patient care and the long working hours they often face (Gün et al. 2024). All of these factors may ultimately lead to prolonged hospitalisations, which, in turn, can increase risks to patient safety and result in higher costs (Galanis, Moisoglou, et al. 2024).

According to Anand et al. (2023), although turnover intention and quiet quitting share similarities, quiet quitting differs from voluntary turnover and work withdrawal. While it involves psychological distancing through reduced output, low engagement, and satisfaction, quiet quitting does not imply an intent to leave one's position. Although there are some different definitions, turnover intention refers ‘the desire of an employee to quit their current job within a certain time period’ (Tolksdorf et al. 2022). Turnover intention in nurses is an important issue in terms of increasing working hours, nurse turnover costs, financial costs, loss of trained employees and affecting service performance (Takase 2010). Nurse turnover is an issue that has plagued many healthcare institutions for years (Labrague et al. 2020). Increasing turnover intention among nurses COVID‐19 pandemic‐related factors, patient–physician‐related factors, psychosocial factors are also effective. Evidence suggests that, high turnover rate in nurses causes decrease in the services, patient safety and quality of services that nurses provided (Gebregziabher et al. 2020; Hou et al. 2021). Turnover intention which occurs due to unsatisfactory, stressful working environments and work overload has adverse outcomes to the healthcare institutions (Yildiz et al. 2021) and the rate of turnover intention among nurses is considerably high among nurses (Gebregziabher et al. 2020; Galanis, Moisoglou, et al. 2024). Nurses, in particular, have faced great challenges such as burnout, depression, anxiety and post‐traumatic stress during the pandemic as they are front‐line healthcare workers (Galanis et al. 2023a). Therefore, nurses experience a high level of turnover intention (Galanis et al. 2023a). In addition, It is believed that individuals wishing to remain in the sector will explore alternative roles, either within the same field or in different professions, that offer reduced working hours and lighter workloads (Formica and Sfodera 2022). On the other side, being a quiet quitter would be an option for nurses.

Nurses are one of the groups most affected by COVID‐19 among healthcare professionals (Yıldırım and Solmaz 2022). The situation is at such a critical level that a study conducted in the United States found that up to 47% of healthcare workers plan to resign from their jobs by 2025, with this rate rising to 90% for nurses (Galanis, Katsiroumpa, et al. 2024). Nurses play a vital role in the delivery of healthcare services. They are expected to be willing to work overtime and remain ready to respond in case of emergencies (Zuzelo 2023). WHO recently declared the healthcare workforce a 5‐year policy priority in the 52nd session of the Executive Board. WHO underlined healthcare workforce shortages in the meeting (WHO 2023). The shortage of nurses is a significant issue in many countries and one of the main challenges faced globally (Takase 2010; Gebregziabher et al. 2020). Moreover, the shortage of healthcare workers (including physicians, nurses, and midwives) was estimated at 7.2 million and is expected to rise to 12.9 million worldwide by 2035 (Gün et al. 2021). The worldwide shortage of nurses is around 6 million (Tolksdorf et al. 2022). One of the main causes of the shortage of nurses is turnover intention. Nurses with turnover intentions who cannot leave their jobs for various reasons also become quiet quitters, which in turn contributes to workforce issues in healthcare (Formica and Sfodera 2022).

Maslach and Jackson (1981) described burnout as a syndrome characterised by emotional exhaustion and cynicism, commonly experienced by individuals engaged in some form of people's work. Burnout is the depletion of psychological resources (Murali et al. 2018). High levels of burnout among healthcare professionals are recognised as a major factor contributing to the widespread occurrence of quiet quitting within the healthcare sector (Kang et al. 2023; Pevec 2023). There are several pieces of evidence that prove job burnout results in negative consequences on nurses' work life. Job burnout is associated with depressive syndromes (Huo et al. 2021), turnover intention (Ran et al. 2020), low level of quality of life (Khatatbeh et al. 2022) and job satisfaction (Acea‐López et al. 2021; Alzailai et al. 2021). Additionally, burnout is one of the most significant predictors of turnover intention (Özkan 2022). Turnover intention and job burnout are associated factors (Ran et al. 2020) and the burnout is a threat to the performance and well‐being of the employees (Özkan 2022). Especially in the pandemic burnout due to long work hours, being far away from families for many days, the infection risk and the psychological harm of uncertainty affected the physical and psychological well‐being of healthcare workers (Yáñez et al. 2020). Nurses are regularly facing multiple types of conflicting situations at the workplace (Kumar et al. 2015) and they are described as frontline fighters and nearly half of the healthcare workers experienced burnout from the beginning of the COVID‐19 pandemic (Ghahramani et al. 2021). Workload, control, reward, community, fairness and values are defined as factors underlying factor of burnout (Maslach 1998; Maslach and Leiter 2016; Maslach et al. 2001; Dall'Ora et al. 2020). As quiet quitting is a novel concept in the literature, there are a limited number of studies on this topic (Gün et al. 2024; Galanis et al. 2023a). To the best of our knowledge, the effect of job burnout on quiet quitting and also the mediating role of turnover intention was assessed among nurses assessed for the first time in the present study.

The aim of the study was to investigate the potential mediating role of turnover intention on the relationship between job burnout and quiet quitting among nurses. Furthermore, this study aimed to shed light on the associations between job burnout, turnover intention and quiet quitting intention. In the light of evidences provided above, the present study aimed to investigate whether turnover intention mediated the effect of job burnout on quiet quitting. In this regard, it is considered turnover intention will be an important factor to explain the underlying mechanism in the relationship between job burnout and quiet quitting. Understanding the underlying mechanism between job burnout, turnover intention and quiet quitting would be useful for both nurses and healthcare administrators. On the basis of the literature job burnout had a significant effect on turnover intention (Ran et al. 2020; Chen et al. 2019) and quiet quitting (Galanis et al. 2023a; Gün et al. 2024). However, to the best of our knowledge, there is no study examined the effect of turnover intention on quiet quitting. In addition, to the best of our knowledge, there were no studies in the literature that explored the direct and indirect effects job burnout on quiet quitting in nurses via job burnout. Considering the gaps mentioned above, this study examined the mediating effect of turnover intention in the relationship between job burnout and nurses' quiet quitting. To end that, we hypothesised that (i) turnover intention and job burnout mean scores differs based on whether nurses are quiet quitters or not. (ii) Job burnout would have direct effect on turnover intention and quiet quitting. (iii) Turnover intention would have direct effect on quiet quitting. (iv) Turnover intention would mediate the association of job burnout with quiet quitting.

## 2 Method

This study was designed as a descriptive, cross‐sectional study. The data were collected between February 2024 and March 2024.

### 2.1 Procedure

In the beginning of the study, the required permissions for using the scales were obtained from the authors via email correspondence. Then, ethical committee approval was received from the Batman University Ethics Committee (Ethic code: 2024/01–04). Informed consent was obtained from the nurses who agreed to participate in the study and they were assured of confidentiality and anonymity of responses. Additionally, the data were collected in accordance with the Helsinki Declaration. The study was conducted with 317 nurses in İstanbul, Türkiye. The inclusion criteria of the study were being over the age of 18 and working in the hospital at least 6 months. A convenience sampling approach was used in the present study due to the time and money constraints. This sampling method has been chosen as nurses work under a high workload, and accessing them, particularly in units such as intensive care, is challenging. This study used paper‐and‐pencil versions of the questionnaires to collect the data. The data of the study were collected between February 2024 and March 2024. The scales take approximately 10 min to complete. Paper‐and‐pencil versions of the questionnaire were applied to nurses who agreed to participate in the study.

### 2.2 Measures

#### 2.2.1 Quiet Quitting (QQ) Scale

The quiet quitting scale was developed by Galanis et al. (2023b). Scale has nine self‐reported items rated on a 5‐point Likert‐type (1 = strongly disagree; 5 = strongly agree). A sample item is ‘I do the basic or minimum amount of work without going above and beyond.’ The scale's internal consistency was 0.80. A higher score obtained from the scale indicates high level of quiet quitting. While quiet quitting scale was used in the first time in Turkish, language validity was assured and confirmatory factor analysis was conducted. Cronbach's alpha value was found to be 0.92 in the present study. According to the confirmatory factor analysis results, indices for the measurement model was CIMIN/DF = 4.42, CFI = 0.97, TLI = 0.96, RMSEA = 0.07 and SRMR = 0.03. The standardised factor loadings ranged from 0.69 (Item 6) to 0.96 (Item 2). Composite reliability of the scale was 0.93 and average variance extracted (AVE) was 0.66. Results showed that there was a good model fit (Hu and Bentler 1999). The results showed that the scale was reliable.

#### 2.2.2 Turnover Intention Scale

The turnover intention scale's reliability and validity conducted by Nadiri and Tanova (2010). The scale was used to measure turnover intentions of the employees. The scale had three self‐reported items and it was rated on a 5‐point Likert‐type (1 = strongly disagree; 5 = strongly agree). Items of the scale were as follows: ‘Often thought of quitting, looking for a new job next year probably, leaving the job next year.’ The Cronbach alpha of the scale was 0.85. In the current study Cronbach's alpha was found to be 0.83.

#### 2.2.3 Job Burnout Scale

To assess individuals' job burnout levels, a job burnout scale was used. The scale that evaluates an individual's physical, emotional, and mental exhaustion levels has 10 self‐reported items rated on a 7‐point Likert‐type (1 never and 7 always) (Pines 2005). Pines (2005) developed the scale and the Turkish validation of the scale was carried out by Çapri (2013). Higher scores refer to higher burnout related to the job. The Cronbach alpha of the scale was over 0.85. In our study, it was 0.96.

### 2.3 Data Analysis

No missing data were detected in the current study. The gathered data were analysed using SPSS (v20) and Process v4.3 for SPSS (Hayes 2017). Percentages of variables, frequency of variables, mean scores of scales and standard deviation were calculated as descriptive statistics. The assumption of normality was controlled by estimating the values of skewness and kurtosis. Prior to testing the mediation model, descriptive statistics, reliability, assumption of normal distribution and correlation coefficients were estimated. Assumption of normal distribution estimated with skewness and kurtosis values ranged between −1.02 and +0.96, based on the rule of thumb of ±2 suggested by George and Mallery (2010) the data normally distributed. Cronbach's alpha coefficient was used to test internal consistency. The independent‐sample *t*‐test was applied to determine the differences in the mean scores of turnover intention and job burnout based on whether nurses are quiet quitters or not. Equality of variance was tested with Levene's test. Confirmatory factor analysis was carried out to the quiet quitting scale to test its construct validity. Pearson's correlation coefficient was used to explore the relationships between the study variables. To test the mediating role of turnover intention in relationship job burnout with and quiet quitting model four was used. The confidence interval was assumed to be 95% at a significance level of *p* < 0.05 and number of bootstrap samples for percentile bootstrap confidence intervals was 5000 (Preacher and Hayes 2008). The results of mediation analysis were evaluated using squared‐multiple correlations (R^2^) and standardised regression estimates (*β*). In the literature effect sizes: 0.01–0.059 referring to small, 0.06–0.139 referring to moderate and 0.14 referring to large (Yıldırım et al. 2023).

## 3 Results

Participants of the present study were composed of 317 nurses who working in a research and training hospital which was a public hospital located in İstanbul, Türkiye. The majority of respondents ranged in between 20 and 31 years old (71.3%) were female (79.5%), single (59.9%) and had a bachelor's degree (59%). An analysis of the hospital units indicates that 34.1% of the participants are employed in hospital clinics, and the rate of those who have been working in the hospital for 1–3 years was 53.9%. Of the participants 49.2% declared that their income was lower than expenditure. A full description of the participants is presented in Table 1.

**TABLE 1:** Demographic characteristics of participants (*N* = 317).

Variable	Number	%
Age		
20–25	100	31.5
26–31	126	39.7
32 and more	91	28.7
Gender		
Female	252	79.5
Male	65	20.5
Marital status		
Single	190	59.9
Married	127	40.1
Education		
Associate degree	74	23.3
Bachelor's degree	187	59.0
Master degree	56	17.7
Unit		
Polyclinic	39	12.3
Clinic	108	34.1
Intensive care unit	17	5.4
Surgical units	22	6.9
Emergency room	40	12.6
Laboratory	91	28.7
Years of working at the hospital		
1–3 years	171	53.9
4 years and more	146	46.1
Income		
Income is lower than expenditure	156	49.2
Income equals expenditure	126	39.7
Income is higher than expenditure	35	11.0
Total	317	100.0

Of the participants, 62.5% were quiet quitters according to the cutoff point of scale (2.06). Using *t*‐test, we also found a significant difference on the mean score of turnover intention (*t* = −4.907, *p* < 0.001) and job burnout life (*t* = −4.660, *p* < 0.001), based on being quiet quitter or not. Mean scores obtained from turnover intention and job burnout were higher in quiet quitters (2.798 ± 1.27; 4.281 ± 1.73, respectively) against none quiet quitters (2.173 ± 0.97; 3.433 ± 1.46, respectively). Additionally, correlation analysis revealed that quiet quitting was positively correlated with turnover intention (*r* = 0.41, *p* < 0.01) and job burnout (*r* = 0.35, *p* < 0.01). Furthermore, there was a positive correlation was found between turnover intention and job burnout (*r* = 0.33, *p* < 0.01) (see Table 2).

**TABLE 2:** Descriptive statistics and correlations between the study variables.

Variable	Mean	SD	Skew	Kurt	*α*	1	2	3
1. Quiet quitting	2.31	0.51	0.71	−0.16	0.92	—	0.41	0.35
2. Turnover intention	2.56	1.20	0.51	−0.71	0.83		—	0.33
3. Job burnout	3.96	1.68	0.14	−1.02	0.96			—

*Note:* All correlations are significant at the 0.001 level (two‐tailed).

**FIGURE 1:**
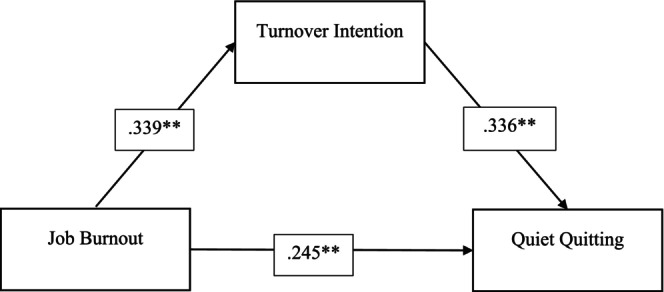
Mediation model indicating the mediating effect of turnover intention in the relationship between job burnout with quiet quitting. **Standardised coefficients, *p* < 0.05.

Prior to the mediation analysis, the direct effects between job burnout, turnover intention and quiet quitting were assessed and the standardised coefficients were presented in Figure 1. The direct relationship findings revealed that job burnout had a significant predictive effect on nurses' turnover intention (*β* = 0.339, *p* < 0.001) and quiet quitting (*β* = 0.245, *p* < 0.001). Additionally, turnover intention displayed a significant predictive effect on quiet quitting (*β* = 0.336, *p* < 0.001) (see Figure 1). Job burnout explained 11% of the variance of job burnout. Job burnout and turnover intention together explained 22% of the variance of quiet quitting (see Table 3).

**TABLE 3:** Unstandardised coefficients for the mediation model.

Predictor	Outcome			
	*M* (Turnover intention)			
	Coeff.	SE	*t*	*p*
*X* (Job burnout)	0.243	0.038	6.396	0.000
Constant	1.599	0.163	9.768	0.000
	*R* ^2^ = 0.115			
	*F* = 40,920; *p* < 0.01			

Predictor	*Y* (Quiet quitting)			
	Coeff.	SE	*t*	*p*
*X* (Job burnout)	0.074	0.016	4.650	0.000
*M* (Turnover intention)	0.142	0.022	6.368	0.000
Constant	1.650	0.074	22.254	0.000
	*R* ^2^ = 0.228			
	*F* = 46.478; *p* < 0.01			

*Note:* Number of bootstrap samples for percentile bootstrap confidence intervals: 5000.

Abbreviations: Coeff, unstandardised coefficient; M, mediator variable; SE, standard error; X, independent variable; Y, outcomes variable.

Mediation analyses were carried out to figure out whether turnover intention mediates the relationship between job burnout and quiet quitting. The indirect effect of job burnout on quiet quitting (*β* = 0.034, 95% CI [0.019, 0.054]) through turnover intention was significant. The level of the indirect effect was positive and significant since the BootLLCI and BootULCI values did not contain zero. The results indicated that turnover intention partially mediated the relationship between job burnout and quiet quitting. Turnover intention plays a mediating role in the effect of job burnout and quiet quitting, and turnover intention is a significant variable in the relationship between job burnout and quiet quitting. Total effect, direct effect and indirect effect are presented in Table 4.

**TABLE 4:** Unstandardised and standardised total, direct and indirect effects of job burnout on quiet quitting.

Effect	Path					Effect	SE	BootLLCI	BootULCI
Total effect	Job burnout	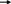	Quiet quitting			0.109	0.16	0.077	0.140
Direct effect	Job burnout	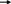	Quiet quitting			0.074	0.016	0.043	0.106
indirect effect	Job burnout	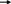	Turnover intention	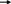	Quiet quitting	0.034	0.009	0.019	0.054
Standardised indirect effect	Job burnout	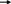	Turnover intention	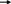	Quiet quitting	0.113	0.028	0.064	0.175

## 4 Discussion

This study investigated the mediating role of turnover intention on the relationship between job burnout and quiet quitting and the associations among job burnout, turnover intention and quiet quitting. Job burnout positively affected both turnover intention and quiet quitting. Additionally, turnover intention was a predictor of quiet quitting intention among nurses. Furthermore, turnover intention partially mediated the relationship between job burnout and quiet quitting. The impact of job burnout on quiet quitting occurs in part through turnover intention which serves as a mediator role in this relationship.

As far as we know, limited studies exist on quiet quitting. To date, to the best of our knowledge, no study examined the mediation role of turnover intention in the relation between job burnout and quiet quitting. Following literature review, this study proposed four main hypotheses. Based on the direct and indirect relationships we proposed, the findings validated all hypotheses. Additionally, the result of the correlation analysis indicated that quiet quitting was positively influenced by turnover intention and job burnout. Furthermore, the results of the present study indicated that job burnout and turnover intention were positively associated variables. As job burnout increases, turnover intention also increases. In support of these findings, earlier studies found that job burnout showed a significant positive correlation between turnover intention and burnout (Ran et al. 2020; Chen et al. 2019).

In the present study, of the participants, 62.5% of nurses were quiet quitters. Consistent with earlier research, based on longitudinal data collected by Gallup at least 50% of the US workforce is represented by quiet quitters in the second quarter of 2022 (Formica and Sfodera 2022). In our study rate of quiet quitters was found 62.5%. Additionally, mean score of quiet quitters on the job burnout and turnover intention scales were also higher than non‐quiet quitters. In the literature, as far we know there was no study that examined mean score differences on job burnout and turnover intention based on being quiet quitter or not. On the basis of the results, the first hypothesis was validated.

Our research findings confirmed the hypothesis that job burnout would have a direct effect on turnover intention. The association between job burnout and quiet quitting was also examined in a limited number of studies. Present study demonstrated that positive and significant relationship between job burnout and quiet quitting. Moreover, job burnout was an important predictor of quiet quitting. In parallel with our findings, in a study conducted by Lu et al. (2023), there was a positive correlation between these variables and they stated that job burnout was a predictor of quiet quitting. In a novel study conducted by Gün et al. (2024), job burnout had a positive effect on quiet quitting intention. As job burnout increases, quiet quitting intention also increases. Furthermore, according to Galanis et al. (2023a) study, findings of multivariable analysis showed a positive relationship between burnout and quiet quitting as in our study. In a study conducted among Chinese Gen Z employees, factors that affect quiet quitting intention were assessed. Based on the findings, job burnout had a significant positive influence on China's Gen Z employees' quiet quitting decision (Xueyun et al. 2023). These results are in parallel with our results. On the other hand, there is a substantial number of research indicating that job burnout significantly impacts turnover intention among nurses. (Lestari and Amalia 2024; Qu et al. 2023; Hassan et al. 2023). The findings of this studies in the literature, similar to our research, confirm that job burnout affects turnover intention and that individuals experiencing job burnout are more inclined towards turnover intention.

There is a gap in the literature that examines the relationship between turnover intention and quiet quitting. Our findings prove that the relationship between turnover intention and quiet quitting was positive and significant. The effect of turnover intention on quiet quitting was significant and the correlation between variables was positive. On the basis of the results, third hypothesis was accepted. In the literature, it was stated employee dissatisfaction and disengagement are additional factors contributing to quiet quitting. These factors are also known to impact employees' turnover intention (Anand et al. 2023). While some studies in the literature suggest that the intention of quiet quitting influences turnover intention (Galanis, Moisoglou, et al. 2024; Kim and Sohn 2024), the quiet quitting literature indicates that individuals with turnover intention who are unable to resign for various reasons may exhibit quiet quitting behaviour (Formica and Sfodera 2022; Scheyett 2023). On the basis of literature, this study suggests that turnover intention affected quiet quitting intention of nurses. The novelty of the concept of quiet quitting contributes to the ambiguity of the exact nature of this relationship. Accordingly, the present study adds valuable evidence to the literature.

The subsequent aim of this research was to investigate the mediating role of turnover intention in elucidating the underlying mechanism between job burnout and quiet quitting intention of nurses. To the best of our knowledge, this is the first study that investigate mediator role of turnover intention in relationship between job burnout and quiet quitting. The results typically confirmed the associations between job burnout, turnover intention, and quiet quitting. Nurses who experience job burnout are significantly associated with turnover intention and quiet quitting. Results of the present study indicated that turnover intention partially mediated the association between job burnout and quiet quitting. In other words, job burnout was positively correlated with turnover intention, which was, in turn, positively correlated with quiet quitting. Therefore, the fourth hypothesis was validated. In parallel with our research, significant associations were found between organisational support, job burnout and quiet quitting. In this study job burnout was partially mediated the relationship organisational support with quiet quitting (Gün et al. 2024).

### 4.1 Implications of the Findings

#### 4.1.1 Theoretical Implications

As the concept of quiet quitting has just emerged in the last quarter of 2022, this study makes a significant contribution to the quiet quitting literature by examining the mediating role of turnover intention in the effect of job burnout on quiet quitting. The results of the research reveal that both job burnout and turnover intention influence quiet quitting behaviour. Furthermore, the study highlights that addressing the direct effect of job burnout on quiet quitting is insufficient, as turnover intention also plays a role in this relationship. In other words, focusing solely on job burnout may not be adequate to reduce quiet quitting intention. Through exploring the interconnections between these factors, this study demonstrates that quiet quitting is not merely an outcome, but a complex phenomenon influenced by variables like job burnout and turnover intention. This offers a fresh insight into the existing literature. The originality and significance of this study are highlighted by its demonstration of how job burnout and turnover intention influence quiet quitting behaviours among nurses working under high workloads. The findings of this study emphasise the importance of turnover intention as a key factor in understanding the relationship between job burnout and quiet quitting, especially in demanding work environments.

#### 4.1.2 Practical Implications

Our model that we tested has important practical implications for line managers and administrators. It is anticipated that addressing the factors contributing to nurses' turnover intention and job burnout will result in a reduction of quiet quitting behaviours. Thus, mitigating the factors that negatively impact the mental and physical well‐being of nurses will facilitate their retention within the organisation.

The findings of this study provide valuable insights for managers. Focusing solely on job burnout may not be sufficient to control nurses' quiet quitting intentions. In addition, turnover intention should also be assessed and considered. Nurses who do not exhibit quiet quitting tendencies are expected to demonstrate higher quality of care, better motivation, and greater job satisfaction compared to others. Furthermore, developing strategies to enhance nurses' motivation may improve the quality of care they provide to patients, thereby enhancing the overall quality of healthcare services. Understanding the interactions between job burnout and turnover intention is a crucial step in addressing workforce challenges in the healthcare sector. The findings of this study suggest that better support for healthcare workers is necessary, which could, in turn, enhance workforce productivity.

### 4.2 Limitation

As with many studies, this study also has some limitations that should be acknowledged. First, the present study designed as cross‐sectional research. Researches in the future on quiet quitting should be conducted on longitudinal design to assess causal link between the variables. Second, the data collected based on self‐reports of nurses that is susceptible to the biases and limitations inherent in self‐reporting. In future researches self‐reporting biases and limitations issues should be considered. Another limitation of the current study is it was conducted only in one hospital. Therefore, the data's findings represent self‐reports of nurses that are working in the same hospital so generalisation of the findings may not be possible. Yet another limitation of the study was the study used convenience sampling method resulted in a greater inclusion of easily accessible nurses in the study. Furhermore, the study conducted among nurses. Therefore, it cannot be generalised to all healthcare workers. Lastly, we examined effects of two variables on quiet quitting.

## 5 Conclusions

The findings obtained from the research contain significant insights for organisational practices and policymakers in the healthcare sector, particularly concerning nurses. Our findings highlight the substantial impact of job burnout on quiet quitting intention, while also indicating that turnover intention should not be overlooked in this relationship. The findings obtained from the present study should be taken into account in management and strategy development processes. They provide important evidence that job burnout, which influences quiet quitting intention, should not be considered alone and that other factors, such as turnover intention, should also be considered. It is crucial for managers to establish policies and develop strategies aimed at reducing job burnout. Another important point is to identify the factors influencing nurses' turnover intention and to create practices, policies and strategies that encourage their retention within the organisation. These measures could be highly beneficial in facilitating a reduction in nurses' quiet quitting intentions. Another important aspect is improving nurses' working conditions, balancing their workloads, particularly in Turkey, increasing and diversifying the healthcare workforce, improving economic conditions and providing social and psychological support to address their issues. These practices could help eliminate job burnout, turnover intention, and, consequently, quiet quitting intentions among nurses. Improving nurses' job well‐being will directly impact the quality of the healthcare and care they provide, leading to a reduction in healthcare costs, more effective service delivery, and consequently, an increase in patient satisfaction in return.

Although the subject of quiet quitting has received a lot of attention, the number of studies on quiet quitting in the literature is quite limited. This study is one of the first studies to investigate the reasons behind quiet quitting intention and to identify problems that may be encountered in the provision of health services by following this in nurses. It is thought that this study will also contribute to the field of human resources management, nursing management and organisational behaviour. The intention of quiet quitting has the potential to profoundly impact and reshape the workforce and there is a big gap in the literature on quiet quitting. Therefore, it is essential to conduct a deeper exploration of this phenomenon, and further studies on the subject should be encouraged. It may be useful to examine the effects of other variables (work overload, change fatigue, income level, job satisfaction, organisational support, organisational culture, etc.) on quiet quitting in further studies.

## Author Contributions

Study conception and design: All authors. Data collection: F.Ç.K. Data analysis and interpretation: İ.G. and S.S. Drafting of the article: All authors. Critical revision of the article: All authors.

## Ethics Statement

The study was approved by the Batman University Ethics Committee (REF:2024/01–04), and the work was conducted in accordance with the Declaration of Helsinki.

## Conflicts of Interest

The authors declare no conflicts of interest.

## Peer Review

The peer review history for this article is available at https://www.webofscience.com/api/gateway/wos/peer‐review/10.1111/jan.16807.

## Data Availability

Data analysed during the current research can be obtained from the corresponding author upon reasonable request.
